# Autism and duplication of 17q12q21.2 by array-CGH: a case report

**DOI:** 10.1590/1984-0462/2023/41/2021387

**Published:** 2023-01-20

**Authors:** Alana Weingartner, Naiara Bozza Pegoraro, Rie Tiba Maglioni, Isabelle Caroline Fasolo Normandia Moreira, Gabriela Esmanhoto Rodrigues, Ana Clara Kunz, Caroline Brandão Piai, Aline Sauzem Milano, Salmo Raskin, Lilian Pereira Ferrari, Liya Regina Mikami

**Affiliations:** a Centro Universitário Autônomo do Brasil, Curitiba, PR, Brazil.; b Faculdade Evangélica Mackenzie do Paraná, Curitiba, PR, Brazil.; c Universidade Federal do Paraná, Curitiba, PR, Brazil.; d Faculdades Pequeno Príncipe, Curitiba, PR, Brazil.; e Pontifícia Universidade Católica do Paraná, Curitiba, PR, Brazil.; f Genetika – Centro de Aconselhamento e Laboratório de Genética, Curitiba, PR, Brazil.

**Keywords:** Autism spectrum disorder, Genetic, Genes, Transtorno do espectro autista, Genética, Genes

## Abstract

**Objective:**

Autism spectrum disorder (ASD) affects cognitive development and social interaction on different levels. Genetic and environmental factors are associated with secondary ASD. Genetic inheritance is mainly polygenic, and 10% are copy number variations (CNVs). Array comparative genomic hybridization (array-CGH) is used to identify CNVs. This report aimed to discuss autism spectrum disorder and its diagnosis by array comparative genomic hybridization, highlighting the association with the pathogenic duplication of 17q12q21.2.

**Case description:**

A male baby was born at 37 weeks’ gestation by cesarean section. The child showed strabismus, cryptorchidism, hypertelorism, frontal bossing, and developmental delay, walking at 25 months and talking at 4 years. At the age of 2 years, array-CGH of peripheral blood revealed a 5.6-Mb 17q12q21.2 duplication or arr 17q12q21.2 (34,815,527-40,213.109)x3 encompassing 190 genes, including *HNF-1B* and *LHX1*. The child was clinically diagnosed with ASD.

**Comments:**

Changes in the 17q12 segment, such as the duplication found, have been associated with the development of several problems in previous studies, mainly kidney diseases and behavioral disorders. Located at this chromosome region, HNF1's homeobox B codes a member of the superfamily containing homeodomain of transcription factors. Another gene associated with abnormalities in neurological development regarding 17q12 deletions is *LHX1*, as shown in this case study. *LHX1* plays a role in the migration and differentiation of GABA neurons, modulating the survival of pre-optical interneurons, thus affecting cellular migration and distribution in the cortex. Changes in this control result in flaws in interneuron development, contributing to the pathophysiology of psychiatric diseases.

## RESUMO

**Objetivo:**

O transtorno do espectro autista (TEA) afeta o desenvolvimento cognitivo e a interação social em diferentes níveis. Fatores genéticos e ambientais estão associados a TEA secundário. A herança genética é principalmente poligênica e, em 10%, são variações do número de cópias (CNV). A hibridização genômica comparativa de *array* (array-CGH) é usada para identificar CNV. Este relato objetivou discutir o TEA e seu diagnóstico por array-CGH, destacando a associação com a duplicação patogênica do 17q12q21.2.

**Descrição do caso:**

Um bebê do sexo masculino nasceu com 37 semanas de gestação por cesariana. A criança apresentou atraso no desenvolvimento, andando aos dois anos e um mês e falando aos quatro, exibindo estrabismo, criptorquidia, hipertelorismo e saliência frontal. Aos dois anos, o array-CGH de sangue periférico revelou duplicação de 5,6 Mb 17q12q21.2 ou arr 17q12q21.2 (34.815.527-40.213.109) x3 abrangendo 190 genes, incluindo *HNF1B* e *LHX1*. A criança foi clinicamente diagnosticada com TEA.

**Comentários:**

Alterações no segmento 17q12, como a duplicação encontrada, têm sido associadas ao desenvolvimento de patologias renais e distúrbios comportamentais. Localizado nessa região cromossômica, o *HNF1B* codifica um membro da superfamília que contém o domínio dos fatores de transcrição. Outro gene foi associado a anormalidades neurológicas, em relação às deleções 17q12, o *LHX1*, como mostrado neste caso. O *LHX1* desempenha um papel na migração e diferenciação dos neurônios GABA, modulando a sobrevivência dos interneurônios pré-ópticos e afetando, assim, a migração e distribuição celular no córtex. Mudanças nesse controle resultam em falhas no desenvolvimento dos interneurônios, contribuindo para a fisiopatologia das doenças psiquiátricas.

## INTRODUCTION

Autism is a multifactorial disease, with several evidences of genetic basis. It is a complex disorder of multiple etiologies and degrees.^1^ Autism spectrum disorder (ASD) can affect a person's cognitive and social development on different levels. Social interactions as well as communication could be compromised, leading to stereotypic behavior and affecting the patient's brain function. Speech and social interaction problems may also be observed, depending on the severity of ASD.^2^ Autism was first described in 1943. After 23 years, the first epidemiological study on autism estimated a prevalence of 4.5 per 10,000 people.^3^ This prevalence has increased significantly throughout time. At present, the prevalence is 1 per 44 children, as stated by the Centers for Disease Control and Prevention (CDC).^4^ According to the *Diagnostic and Statistical Manual of Mental Disorders* (*DSM-5*), only the following two areas are in major diagnostic criteria for ASD: (1) persistent social communication and interaction deficits in various contexts and (2) behavior, interests, or activities that show repetitive and restrictive patterns.^3^

The study genes and their involvement in the diagnosis of ASD can be essential to its understanding and, consequently, to the early diagnosis and appropriate treatment for patients with ASD. There are many concrete evidences of the genetic role in the disease's pathophysiology. Despite that, its etiology is of great complexity and involves different genes in different chromosomes — it may even be considered that almost all chromosomes have been at some point correlated to ASD.^2^

Considering that ASD is vast and broad, its diagnosis usually happens simultaneously with other diseases, allowing the identification of genetic modifications that correlate to the patient's clinical presentation. ASD is considered a disease of complex interaction between genes and environment, with an estimated genetic inheritance of 40–80%. Genetic studies have already identified hundreds of genes related to autism, and individuals with similar pathogenic variants may vary drastically when it comes to phenotype. Factors that modulate genes’ expression or genetic modifiers are most likely present in patients with opposite disease spectra. Thence, this genetic inheritance may be polygenic and have different genetic modifiers — 10% of which are copy number variations (CNVs), in addition to double-hit mutations, epigenetic influence, and sex-related causes.^3^

Array comparative genomic hybridization (array-CGH) is used to identify CNVs. Technological advances in chromosome studies through microarray allowed identification of the prevalence of CNVs. Therefore, the chromosome microarray analysis is an extremely important tool for ASD patients’ clinical investigation. Nevertheless, the alterations’ clinical interpretation can be challenging due to the patients’ broad phenotypic spectrum. One explanation for these inconsistencies is the complex interactions between potentially pathogenic CNVs and the gender or environmental factor specific to each case report.^5^

Despite the lack of studies in the literature showing patients with duplication of the 17q12 segment, 12 cases were reported by Miloni et al., in which patients were gathered in a period of 10 years at the Stella Maris Institute.^6^ The patients were then evaluated and showed neuropsychiatric syndromes, which helped setting the diagnosis, as well as visceral malformations.^6–8^ The few other existent cases describe a phenotype that includes motor and speech development delay, learning difficulties associated with a wide spectrum of psychiatric and neurological disorders,^8^ as well as renal, cardiac, and ocular modifications.^7^ Thus, a diversity of clinical manifestations is noticeable in individuals with 17q12 segment duplication, which can be attributed to aspects such as incomplete penetrance, environmental and epigenetic factors, genetic modifiers, and disparity in duplicated segment sizes.^9,10^ The estimated prevalence of 17q12 duplication, on a Danish epidemiological study was that of 4.5 in 1,000,000 people.^8^ The purpose of this article was to discuss ASD's diagnostics and 17q12 segment duplication — identified in the described patient through array-CGH genetic examination — aiming to stress out the benefits and necessity of genetic counseling during investigation of patients with development delay.

## CASE REPORT

A male baby was born through a cesarean section at 37 weeks’ gestation. The baby's first pediatric appointment took place at the age of 1 year, at which point the mother was 27 years old and the father was 46 years old. The patient's medical record and phenotype indicated a speech and motor development delay, walking for the first time at age 2 and 1 month, and first signs of speech at the age of 4 years. Other identified signs were strabismus, cryptorchidism, hypertelorism, and a frontal bossing. At the time, the patient's father reported having a 9-year-old daughter from another relationship, who did not show any signs of development delay. The diagnostic hypothesis of ASD was deduced from the patient's clinic and confirmed by microdeletion examination by FISH. At the age of 2 years, array-CGH of peripheral blood revealed a 5.6-Mb duplication of 17q12q21.2 or arr 17q12q21.2(34,815,527-40,213.109)x3 encompassing 190 genes, including *HNF1B* and *LHX1*.

## DISCUSSION

This case report contributes to the search for information about the genetic profile of ASD through array-CGH and proposes further discussions in regard to the possible association between ASD and the pathogenic 17q12q21.2 locus duplication, by associating the findings on the case report with previous described literature.

Since the early 2010s, the American Academy of Child and Adolescent Psychiatry as well as the American Academy of Pediatrics recommend the use of genetic examinations for investigation of patients’ idiopathic neurocognitive development delays.^9^ The greater dissemination of these examinations allowed many genetic alterations to be identified and correlated with the patient's phenotype, making the treatment for this disease possible. Despite the access to these different tests being easier today, it remains hard to evaluate the pathogenic potential of different CNVs and their influence on the phenotype.^10^

In recent literature, duplications of the 17q12 segment are associated not only with ASD but also with structural central nervous system abnormalities, learning difficulties, epilepsy, schizophrenia, facial dimorphism, kidney diseases, anal and esophageal atresia, ligament laxity, and endocrine disorders. Such alterations are due mainly to the impairment of three genes: *HNF1B*, *ACACA*, and *LHX1*.^8,10^

Modifications on the 17q12 segment have already been associated with pediatric kidney disease and *MODY5*-type diabetes. A reciprocal duplication, reported by Mefford et al. in 2007, was correlated with epilepsy. It is known that the *LHX1* gene is involved in the development of the brain and its mutation was found in patients with cortical focal dysplasia and epilepsy. A possible explanation for the development of such brain damaging diseases – including ASD – is the incomplete penetrance in association with the duplication.^11^

Studies have also shown that the *LHX1* gene has a significant part on the migration and differentiation of inhibitory neurons (especially GABAergic). Changes on this gene, as the one seen on this case report, can entail alterations on the position and total number of cells inside the cerebral cortex and the embryonic telencephalon. Its suppression can lead to decrease of neuron quantity in the mentioned regions, because of the repression of pro-survival genes and increase in the pro-apoptotic genes, such as *UNC5B*. Such mutations result in interneuron development failure, contributing to neurological and psychiatric diseases’ pathophysiology.^12^

Another gene affected by the duplication of segment 17q12 is *ACACA* (acetyl-CoA-carboxylase-alpha), which is involved in the metabolism of acetyl-CoA.^10^ Duplications of this gene implicates in the overuse of acetyl-CoA, leading to reduction of acetylcholine and energy synthesis, which could trigger seizures.^13^

Besides neuropsychiatric manifestations, duplications of the 17q12 segment can cause modifications of the *HNF1B* gene locus, which is associated with non-insulin-dependent diabetes mellitus, renal cysts, and renal cell carcinoma. Lastly, it is important to point out that none of the mentioned genes have a well-established causal link to alterations of the 17q12 locus.

The case presented here highlights the importance of genetic evaluation and counseling in patients with a clinical diagnosis of ASD and neurocognitive developmental delay. Array-CGH established the diagnosis of 17q12 segment duplication in our patient, providing another example of the distinct phenotypes associated with this pathogenic duplication.
